# Comparison of Computer-Controlled Intraligamentary Local Anesthesia and Conventional Inferior Alveolar Nerve Block in Primary Molar Extractions: A Randomized Clinical Trial

**DOI:** 10.3390/healthcare14152324

**Published:** 2026-08-01

**Authors:** Lamia AlGhamdi, Osama Felemban, Khlood Baghlaf, Heba M. Elkhodary

**Affiliations:** 1 Pediatric Dentistry Department, AlAhsa Health Cluster, Alahsa 31982, Saudi Arabia; lasalghamdi@moh.gov.sa; 2 Pediatric Dentistry Department, Faculty of Dentistry, King Abdulaziz University, Jeddah 21589, Saudi Arabia; omfelemban@kau.edu.sa; 3 Department of Pedodontics and Oral Health, Faculty of Dental Medicine for Girls, Al Azhar University, Cairo 11651, Egypt; hebaelkhodary.2625@azhar.edu.eg

**Keywords:** anesthesia, dental, pain, extraction, tooth, deciduous

## Abstract

**Highlights:**

**What are the main findings?**

- The findings suggest that interligamentary anesthesia using a Computer-Controlled Local Anesthesia Delivery System provides less painful injections compared to the conventional inferior alveolar nerve block.
- It has demonstrated effectiveness as a viable alternative for the extraction of mandibular primary molars.

**What are the implications of the main findings?**

- By promoting the use of CCLADS, pediatric dentists can address the negative behaviors children may exhibit towards dental treatment, fostering a more positive dental environment and encouraging future visits.
- The findings provide valuable clinical evidence supporting the adoption of advanced anesthesia techniques, enabling pediatric dentists to make informed decisions that enhance care quality and patient satisfaction.

**Abstract:**

**Background/Objectives:** The study evaluates pain perception in children aged 5–9 during dental extractions, comparing intraligamentary anesthesia (ILA) delivered with a Computer-Controlled Local Anesthetic Delivery System (CCLADS) vs. the traditional inferior alveolar nerve block (IANB). **Methods:** A randomized clinical trial with 60 healthy, cooperative children measured pain both subjectively using the Wong–Baker Faces Pain Rating Scale (WBFPRS) and objectively using the Sound-Eye-Motor (SEM) scale. **Results:** Results showed significantly lower mean pain scores with CCLADS during anesthesia administration: WBFPRS was 2.0 ± 2.2 vs. 6.9 ± 2.6 for IANB (*p* < 0.001). Objective SEM scores during anesthesia delivery were also lower with CCLADS (3.7 ± 0.8) compared with IANB (5.7 ± 1.2) (*p* < 0.001). During the extraction itself, SEM scores did not differ significantly between groups. **Conclusions:** The authors conclude that ILA via CCLADS reduces discomfort during anesthesia without compromising anesthetic effectiveness.

## 1. Introduction

The application of local anesthesia (LA) in dentistry has significantly minimized the pain and discomfort typically associated with dental procedures [1]. Various techniques have been proposed to reduce pain and discomfort during needle insertion and the delivery of anesthetic solutions. However, the manual control of syringe pressure and needle vibration can be technically challenging [1,2,3]. Currently, the Computer-Controlled Local Anesthetic Delivery System has emerged as an effective method for reducing pain during local dental anesthesia administration [4].

The primary objective of CCLADS is to deliver local anesthetic solutions in a precise, controlled manner, minimizing injection pain and side effects often associated with conventional techniques [5,6]. This system maintains a consistent flow, administering one drop of anesthetic every two seconds regardless of tissue density [4,7]. In 2006, a newer device emerged, specifically designed for dental applications, the Single Tooth Anesthesia System (STA System) (Milestone Scientific, Inc., Livingston, NJ, USA). The STA System delivers local anesthetic precisely and accurately to a single tooth via the intraligamentary route [8,9]. It offers advantages over its predecessors, including a training mode that provides audible guidance on device usage. However, the system has drawbacks, including high cost, complexity, the need for storage space, and the extended time required for local anesthetic administration [7]. Also, some anesthetic volume may be lost in CCLADS due to technical factors, which can affect the onset, pain control, and duration of anesthesia [10].

The literature reveals that CCLADS offers the added benefit of being a more child-friendly technology. Several studies indicated that ILA using CCLADS was superior to conventional LA syringes [1,8,11,12,13,14], while other studies indicated no difference between the two techniques [12,15,16]. Moreover, few clinical trials have evaluated ILA using CCLADS for the extraction of mandibular primary molars. The achievement of effective local anesthesia is a key component of dental care, particularly for tooth extraction procedures, where sufficient pain management is vital for treatment success and a positive patient experience. Effective local anesthesia is crucial during tooth extractions, as it ensures adequate pain control, contributes to successful treatment, and improves patient comfort. To date, only Helmy et al. [6] and Prol Castelo et al. [17] have investigated the effectiveness of intraligamentary anesthesia (ILA) delivered using a Computer-Controlled Local Anesthetic Delivery System (CCLADS) for the extraction of primary molars. Although these studies reported favorable outcomes, they used different pain assessment methods, limiting direct comparison of their findings. Furthermore, Elbay et al. [18] reported conflicting results regarding the effectiveness of this technique. Consequently, the available evidence remains limited and inconclusive, and there is a lack of standardized clinical studies evaluating both pain perception during injection and anesthetic efficacy in children undergoing primary molar extraction. Therefore, this study aimed to compare pain perception during the administration of ILA using CCLADS with conventional inferior alveolar nerve block (IANB) and to evaluate the anesthetic effectiveness of both techniques during primary molar extraction in children aged 5–9 years. The null hypothesis was that there would be no significant differences between intraligamentary anesthesia (ILA) delivered using a Computer-Controlled Local Anesthetic Delivery System (CCLADS) and the conventional inferior alveolar nerve block (IANB) with respect to pain perception during anesthetic administration and anesthetic effectiveness during primary molar extraction in children aged 5–9 years.

## 2. Materials and Methods

### 2.1. The Study Design

The study was a randomized, parallel, clinical trial that was conducted in the Pediatric Dentistry Department, University Dental Hospital. Recruited children were randomly assigned based on the local anesthesia technique employed, as follows: ILA using the CCLADS group and the IANB group.

### 2.2. Ethical Consideration

Ethical approval was obtained from the Research Ethics Committee of the Faculty of Dentistry, under approval number (109-05-23). The study protocol was registered in the clinical trials registry (https://clinicaltrials.gov/), under the identifier number (NCT066690392).

### 2.3. Sample Size Estimation

To determine the appropriate sample size for the study, the expected findings of the Wong–Baker Faces Pain Rating Scale (WBFPRS) and the Sound-Eye-Motor (SEM) scale were taken into consideration based on a previous study [6]. It was determined that a sample size of 30 patients in each group would provide 95% power for an independent sample *t*-test or Mann–Whitney U test to detect a difference in means or medians of WBFPRS and SEM scale of 2 between groups, assuming a common standard deviation of 2.1 at the 0.05 significance level.

### 2.4. Eligibility Criteria

Patients between the ages of 5 and 9 years who required extraction of their mandibular first primary molar were eligible for the study. These included healthy patients demonstrating cooperative behavior and classified as “definitely positive” according to the Frankl behavior classification scale [19]. Patients who had facial cellulitis or consumed pain medication within 6 h before their scheduled dental appointments were excluded. The subjects had to have a mandibular first primary molar planned for extraction due to extensive caries, furcation involvement, or unsuccessful pulp treatment. The first primary molars were excluded if they had grade III mobility, ankylosis, root resorption (more than 1/3 of the total root length), severe provoked pain, gingival abscess, tenderness to percussion, or fistula. A written informed consent for the child’s participation was obtained from the parent (or guardian) after explaining the objectives, risks, and benefits of the study.

### 2.5. Randomization, Allocation Concealment and Blinding

After being enrolled in the study, each subject was then randomly assigned to a group by being asked to select a closed envelope from a bowl held by the primary investigator. All 60 envelopes were identical in size and color to ensure allocation concealment from the operator. Following enrollment, participants were randomly allocated to one of the two study groups using a simple randomization procedure with a 1:1 allocation ratio. The random allocation sequence was generated prior to participant recruitment by an independent investigator who was not involved in patient recruitment, treatment administration, or outcome assessment. Group assignments were placed in 60 sequentially numbered, opaque, sealed envelopes (30 assigned to the intraligamentary anesthesia [ILA] using the CCLADS group and 30 assigned to the conventional IANB group). Blinding was not possible because the two anesthesia techniques differ both visually and technically.

### 2.6. Intervention

To standardize procedures and minimize variability, anesthesia administration techniques and extraction procedures in both groups were performed exclusively by a single trained, calibrated pediatric dentist (primary investigator) who was calibrated on the local anesthesia administration with the ILA using CCLADS and also with the IANB. Once the anesthesia technique was assigned, the parents were informed of their child’s group. The treatment was then explained to the child using simple, age-appropriate language, and the child’s behavior was managed using various non-pharmacologic basic behavior guidance.

- **ILA using CCLADS Technique**

Topical anesthetic gel (20% benzocaine) was applied at the lingual alveolar ridge of the extraction site using a cotton swab for 2 min, covered by 2 × 2 gauze. The ILA using CCLADS anesthesia was administered using the STA^®^ system (Milestone Scientific Inc., Roseland, NJ, USA), connected to a disposable 30-gauge (half-inch) ultra-short needle with a 1.8 mL local anesthetic cartridge containing 2% Lidocaine HCl with 1:100,000 epinephrine. The needle was then inserted into the crevice between the gum and tooth, starting at the disto-lingual line angle at approximately a 30° angle relative to the longitudinal orientation of the tooth, with the needle bevel facing the tooth structure. The needle was gently advanced into the sulcus until the periodontal ligament resistance was detected and the pressure-sensing indicator illuminated a green light and announced “PDL,” confirming the correct position and applied pressure. After about a quarter of the carpule was delivered, this process was repeated for the mesio-lingual line angle to deliver the remaining quarter of the carpule [20].

B. **IANB Technique**

According to the IANB injection protocol, a conventional approach was employed where 20% benzocaine topical anesthetic gel was applied using a cotton swab on the pterygomandibular raphe for 2 min while covered by 2 × 2 gauze at the designated injection site utilizing a disposable long 27-gauge dental needle. Precise guidance was initiated from the contralateral side of the dental arch, which was aligned with the occlusal plane. The needle was inserted until resistance from the bone was detected. Subsequently, the needle was retracted by 2 mm to facilitate aspiration, followed by the injection of two-thirds of 2% Lidocaine HCl with 1:100,000 epinephrine. The needle was then inserted at the deepest point of the mucobuccal fold and above the apex of the first primary molar, where a few drops of anesthesia were administered as an infiltration.

### 2.7. Extraction Procedure

Four steps were followed for the extraction procedure: retracting the gingiva first using Molt #9 periosteal elevator, then luxating the tooth by breaking the PDL with a luxation elevator, afterward tooth elevation to loosen the tooth using Coupland elevator, and finally removal of the tooth with lower primary molar forceps. Tooth removal was carried out by applying slow, continuous force from the lingual and buccal sides. Additionally, the mandible was supported to protect the temporomandibular joints from any possible injury. After the extraction was done, the parents were then assured and provided with post-extraction care instructions. Additionally, plans for space maintenance were taken into consideration if needed.

### 2.8. Outcome Assessment

After the anesthesia injection procedure was done, pain perception was assessed subjectively by the child using the Wong–Baker Faces Pain Rating Scale (WBFPRS) and objectively using the Sound-Eye-Motor (SEM) scale. The WBFPRS features 6 facial expressions ranging from an extremely happy face (score of 0) indicating no pain, to an extremely sad face with tears (score of 10) indicating intense pain. This method is commonly used for self-reporting of acute pain in school-aged children [21].

The anesthesia procedure and the extraction procedure were video recorded at various stages with two high-definition cameras recording the patient’s reactions. The anesthesia administration and tooth extraction procedures were video recorded using two high-definition cameras positioned to capture the child’s facial expressions, body movements, and verbal responses from different angles. The videos were later watched by the operator to assess the SEM twice, with a one-week interval between assessments. This operator was calibrated to assess videos using SEM scoring, and the Kappa of intra-examiner reliability was 0.90. One time for the pain during local anesthesia administration and a second time for the pain during the extraction. The SEM is an objective assessment of pain that takes into account the following elements:

1. Eye Movement: Observing involuntary eye movements that may indicate discomfort or pain.
2. Motor Responses: Evaluating how a patient interacts with their environment or responds to stimuli, which can be indicators of pain levels.
3. Verbal and Non-verbal Cues: Integrating patient feedback, whether verbal or through body language, to assess pain severity [22].

### 2.9. Statistical Analysis

Statistical analysis was carried out using the Statistical Package for Social Sciences version (SPSS version 20.0 (SPSS Inc., Chicago, IL, USA), for Windows, Version 20.0 (IBM Corp., Armonk, New York, USA)). Since the WFPRS and the SEM are count data, the Mann–Whitney U test was used for group comparisons. The significance level was set at *p* < 0.05. For Mann–Whitney U comparisons, effect size was reported as rank-biserial correlation (r_rb_). The magnitude of effect was interpreted as small (≈0.10), moderate (≈0.30), or large (≥0.50).

## 3. Results

### 3.1. Descriptive Results

**Figure 1:**
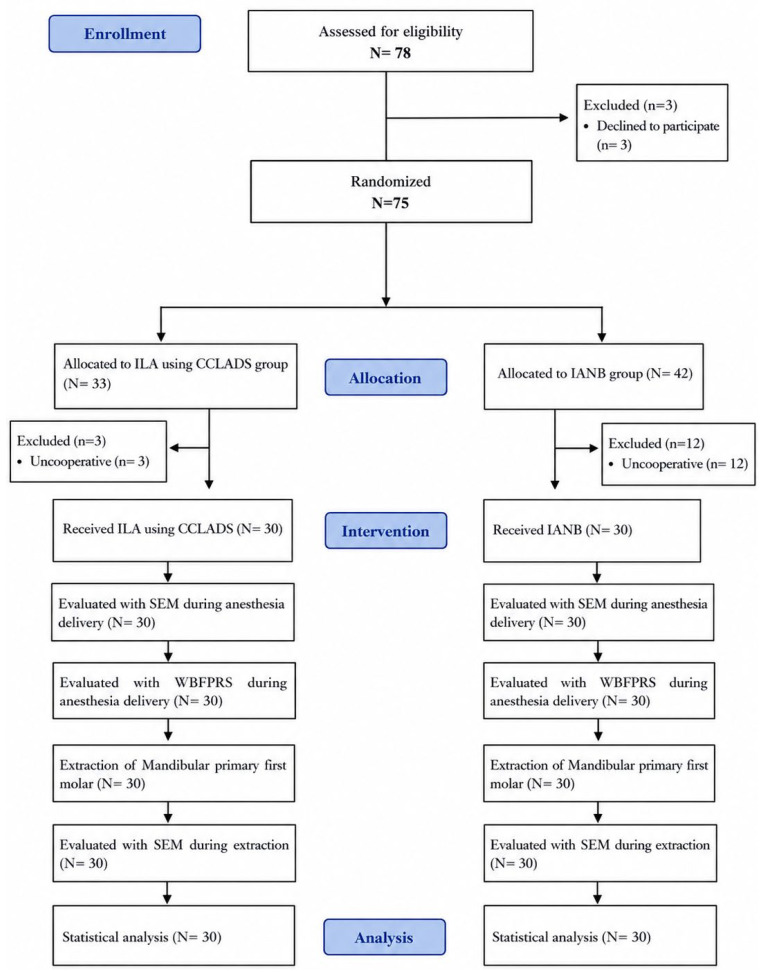
**Flow chart of the sample selection process.** ILA using CCLADS: Computer Controlled Intraligamentary Local Anesthesia. IANB: inferior alveolar nerve block. WBFPRS: Wong–Baker Faces Pain Rating Scale. SEM: Sound-Eye-Motor scale.

In the study, 78 children were identified and assessed for eligibility to participate. However, the parents of three participants declined to participate. Seventy-five subjects were randomized to one of the two groups. Then, 15 subjects exhibited uncooperative behavior on the trial day following randomization and were excluded from the study. Twelve of the fifteen were from the IANB group, and three were from the ILA using CCLADS group. The final sample included 60 subjects, who were randomized based on the local anesthesia technique, with each group comprising 30 subjects (Figure 1).

The mean age in the ILA using CCLADS group was (7.5 ± 1.4) years, while the IANB group had a mean age of (7.4 ± 1.2) years. There was no significant age difference between the two groups (*p* = 0.760). In terms of gender distribution, the ILA using CCLADS group consisted of 10 males and 20 females, while the IANB group included 12 males and 18 females. There was also no significant gender difference between the two groups (*p* = 0.592) (Table 1).

#### 3.1.1. Pain Perception

Subjects’ pain perception during LA administration was assessed using the WBFPRS. A significant difference in WBFPRS scores between the two groups was observed (*p* < 0.001), with greater average pain scores in the IANB group (6.9 ± 2.6) compared to the ILA using CCLADS group (2.0 ± 2.2). The operator’s evaluation of pain during anesthesia injection was measured using the SEM scale. A significant difference in SEM scores was also observed (*p* < 0.001), with higher mean pain scores in the IANB group (5.7 ± 1.2) compared to the ILA using CCLADS group (3.7 ± 0.8) (Table 2).

#### 3.1.2. Anesthesia Effectiveness During Extraction

For the various steps of tooth extraction, the mean SEM during gingival retraction was 3.0 ± 0.2 in the ILA using CCLADS and 3.0 ± 0.0 in the IANB group. During tooth luxation, the ILA using CCLADS group had a mean SEM of 3.4 ± 0.7, compared to 3.6 ± 0.7 for the IANB group. For tooth elevation, the ILA using CCLADS group had a mean SEM of 4.3 ± 1.1, while the IANB group recorded 4.1 ± 0.8. Finally, during tooth removal, the ILA using CCLADS group had a mean SEM of 3.8 ± 1.0, and the IANB group had a mean of 3.3 ± 0.9 (Table 3). Statistical analysis revealed no significant differences in the SEM scores of the two anesthesia techniques during soft tissue retraction (*p* = 0.317), tooth luxation (*p* = 0.359), tooth elevation (*p* = 0.875), and tooth removal (*p* = 0.051) in the extraction procedure (Table 3).

## 4. Discussion

This randomized clinical trial was conducted to evaluate the pain perception and anesthesia effectiveness of ILA delivered via CCLADS vs. the conventional IANB for primary molar extractions. The main findings of this randomized clinical trial showed that local anesthesia administration of the ILA using CCLADS was significantly less painful than the IANB injection. Both ILA using CCLADS and IANB provided effective anesthesia in the extraction of primary molars. The findings contribute to the existing knowledge gap by being one of the few investigations to assess and compare ILA using CCLADS with IANB, specifically in the context of extracting mandibular primary molars.

To reduce potential confounding variables and enhance standardization, only the first primary molar was chosen for extraction in our study. The mandibular first primary molar was selected because it is one of the most frequently treated teeth in pediatric dentistry and commonly requires restorative procedures, making it a clinically relevant and standardized model for evaluating anesthetic efficacy. The intensity of pain experienced in primary first and second molars may vary, even when subjected to similar caries conditions. Notable distinctions were confirmed between the pulp of first and second primary molars in terms of nerve fiber density and mean diameters [23]. This provides insight into the clinical observation that first primary molars exhibit lower pain sensitivity than second primary molars [23]. Thus, to standardize the reporting of pain, only first primary molars were included in the study.

Only children classified as “definitely positive” according to the Frankl Behavior Rating Scale were included. This criterion was adopted to minimize the potential influence of behavior management difficulties, fear, and anxiety on pain perception and behavioral responses during local anesthesia administration and tooth extraction, thereby reducing potential confounding factors and allowing a more accurate comparison of the anesthetic techniques.

In our study, the WBFPRS was used to measure the children’s perception of pain, while the operator used the SEM scale to rate the pain that the children felt during the anesthesia delivery and extraction procedure. The WBFPRS functions as a subjective scale for pain assessment, allowing children to assign scores to pain sensations through a graded series of joyful and distressed facial expressions [24]. The SEM scale is an objective measurement tool employed in previous research to assess comfort or pain levels in children, and its accuracy has been proven [6]. In our study, heart rate was not employed as a method for pain assessment. Heart rate elevation can result from anxiety or pain, rendering it a secondary and indirect measure of actual pain levels. Additionally, increases in heart rate may occur due to the epinephrine-containing anesthesia solution [25].

In the present study, a local anesthetic cartridge containing 2% Lidocaine HCl with 1:100,000 epinephrine was used in both groups. Both clinical practice and research have reliably confirmed the effectiveness and safety of lidocaine, with low toxicity and rare occurrences of allergic reactions [26]. On the other hand, a meta-analysis comparing the effects and adverse reactions of articaine and lidocaine in pediatric dental procedures found insufficient evidence to support the use of articaine over lidocaine [27].

In terms of pain perception, the present study found that ILA using CCLADS was significantly less painful and better tolerated by young children during LA administration, resulting in less discomfort compared to the conventional IANB. Our findings agreed with previous studies [1,8,11,12,13,14]. In contrast, our findings deviated from the results of other studies [12,15,16], which reported comparable levels of pain perception between CCLADS and conventional techniques. It is worth noting that while Mittal et al. 12 employed a similar objective pain assessment method using SEM in their study, they also included older children with a mean age of 9.14 years. This difference in age groups may account for the observed results, as older children might have an enhanced ability to manage and control their reaction to pain. Alberto et al. [15] investigated a comparable age group to ours (4 to 10 years) using the VAS for pain assessment, but did not specify the mean age of their sample or the age percentage distribution of the children included. Age can be a significant factor, particularly if most participants are older. Moreover, Hachem et al. [16] relied solely on the WBFPRS for pain assessment, which is known for its subjective nature. Additionally, in El Hachem et al.’s study, the children were asked to evaluate their pain experience with anesthesia only after the treatment procedure was completed, which may have impacted their assessment due to the considerable time lapse between anesthesia delivery and the end of the procedure. Also, the subjects may have confused their pain experience during LA with the pain during the dental treatment procedure.

Regarding the effectiveness of anesthesia during extraction procedures, the study showed that ILA using CCLADS is effective for extracting primary mandibular first molars in children aged 5–9 years. Their effectiveness can be attributed to the presence of the Dynamic Pressure Sensing (DPS) feature in the used STA System, which enhances the reliability of ILA, resulting in predictable anesthesia [28]. Our findings are consistent with Castelo et al. [17], who concluded that both ILA using CCLADS and IANB are effective for performing invasive dental procedures in children, including extractions. However, although the technology used to deliver ILA in this study was similar to ours, their evaluation encompassed a wider range of dental procedures, with only 14% being extractions. Additionally, the participants in their study were aged 6–12 years, representing a broader age range than that of our study. Helmy et al. 6 also demonstrated that ILA using CCLADS is equally effective as IANB for primary tooth extractions in children aged 5–7 years. Although their study utilized SEM, the same effectiveness evaluation scale as ours, it is important to highlight that the anesthetic agent used for both ILA using CCLADS and IANB was 4% Articaine HCl with 1:100,000 epinephrine. The increased lipophilicity of Articaine enhances tissue penetration, which may have influenced the evaluation of the actual effectiveness of ILA using CCLADS [26]. In contrast, our findings differed from those of Elbay et al. [18], who examined the effectiveness of ILA vs. infiltration anesthesia, both delivered using CCLADS in dental extractions. Their study concluded that infiltration anesthesia was more effective than ILA during the extraction steps. However, they employed a CCLADS device called the SleeperOne^®^ system, which lacks the Dynamic Pressure Sensing (DPS) feature that aids in delivering consistent ILA. Additionally, their evaluation relied solely on the WBFPRS, a subjective measure by nature. Several clinical trials showed that CCLAD is effective in other dental procedures requiring local anesthesia, including restorative treatment, pulp therapy, extractions, and selected minor soft tissue procedures [13,26].

Our findings are in agreement with a recent systematic review and meta-analysis including 20 randomized controlled trials, which demonstrated that CCLAD effectively reduces injection pain in children, particularly according to subjective pain ratings (Wong–Baker Pain Rating Scale) and physiological responses (heart rate) [29]. However, the review also highlighted inconsistent findings across other pain assessment tools and emphasized the need for further high-quality studies to strengthen the current evidence.

This randomized clinical trial was conducted with a sufficient sample. The methodology was designed to minimize confounding, as patients were randomly assigned to different anesthesia techniques. Additionally, all clinical procedures were performed by a single trained pediatric dentist, ensuring consistency and reliability in the treatment administered.

However, the current study has certain limitations. One significant factor is the potential influence of pre-procedural anxiety, which is common in dental settings and may adversely affect pain assessments through WBFPRS and SEM scales during both the anesthesia injection and extraction procedures. However, even if anxiety was a potential confounder, randomization probably distributed anxious patients equally between groups, canceling any effect of anxiety on pain assessment. The inclusion of only children with a “definitely positive” Frankl Behavior Rating may have limited the external validity of the study, as the results cannot be readily extrapolated to children with negative or less cooperative behavior. The study also did not assess other important variables, such as the time needed to achieve profound anesthesia and the total duration of the procedure for both groups. This outcome may be clinically relevant when comparing anesthetic delivery techniques and should be considered in future studies. Lastly, the research was carried out on a specific population who attended a single center (KAUDH). Therefore, additional studies are necessary to assess the generalizability of the findings to a broader population.

## 5. Conclusions

Based on the results of this study, the findings suggest that interligamentary anesthesia using a Computer-Controlled Local Anesthesia Delivery System provides less painful injections compared to the conventional inferior alveolar nerve block, and it has demonstrated effectiveness as a viable alternative for the extraction of mandibular primary molars. Given the demonstrated effectiveness of interligamentary anesthesia (ILA) using a Computer-Controlled Local Anesthesia Delivery System (CCLADS) in reducing pain during invasive dental procedures, it is recommended to promote the increased use of ILA with CCLADS in dental practices to help mitigate negative behaviors in children towards dental treatment, as well as to provide training for dentists, particularly pediatric dentists, on the integration of CCLADS technology into clinical practice.

This conclusion is limited to the included population, and caution should be exercised when extrapolating these findings to other patient groups or clinical settings. Further well-designed studies involving more diverse populations are needed before broad clinical recommendations regarding the use of CCLAD can be made.

## Figures and Tables

**Table 1:** Descriptive characteristics of the participants (n = 60).

Demographics	Categories	ILA Using CCLADS Group n = 30 n (%)	IANB Group n = 30 n (%)	*p*-Value
**Age**	Mean ± SD	7.5 ± 1.4	7.4 ± 1.2	0.760 †
**Gender**	Male	10 (33.3)	12 (40.0)	0.592 €
	Female	20 (66.7)	18 (60.0)	

† Mann–Whitney U test. € Chi-square test, ILA using CCLADS: intraligamentary anesthesia using Computer-Controlled Local Anesthesia Delivery System, IANB: inferior alveolar nerve block.

**Table 2:** Comparison of child pain perception and operator pain assessment during LA administration.

Measures	ILA Using CCLADS Group (n = 30) Mean ± SD Median (Min–Max)	IANB Group (n = 30) Mean ± SD Median (Min–Max)	*p*-Value †	Effect Size (Rank-Biserial Correlation $r_{rb}$)
**WBFPRS** **during anesthesia**	2.0 ± 2.2 2.0 (0–10)	6.9 ± 2.6 8.0 (0–10)	<0.001 *	−0.81
**SEM** **during anesthesia**	3.7 ± 0.8 4.0 (3–6)	5.7 ± 1.2 5.5 (3–9)	<0.001 *	−0.81

† Mann–Whitney U test. * Statistically significant (*p* < 0.05). ILA using CCLADS: intraligamentary anesthesia using Computer-Controlled Local Anesthesia Delivery System. IANB: inferior alveolar nerve block. WBFPRS: Wong–Baker Faces Pain Rating Scale. SEM: Sound-Eye-Motor scale. Effect size is reported as rank-biserial correlation; positive values indicate higher scores in the IANB group compared with the ILA using CCLADS group, whereas negative values indicate higher scores in the ILA using CCLADS group.

**Table 3:** Comparison of anesthesia effectiveness during extraction steps.

Measures	ILA Using CCLADS Group (n = 30) Mean ± SD Median (Min–Max)	IANB Group (n = 30) Mean ± SD Median (Min–Max)	*p*-Value †	Effect Size (Rank-Biserial Correlation $r_{rb}$)
**SEM soft tissue retraction**	3.0 ± 0.2 3.0 (3–4)	3.0 ± 0.0 3.0 (3–3)	0.317	0.03
**SEM luxation**	3.4 ± 0.7 3.0 (3–5)	3.6 ± 0.7 3.0 (3–5)	0.359	−0.12
**SEM Elevation**	4.3 ± 1.1 4.0 (3–7)	4.1 ± 0.8 4.0 (3–6)	0.875	0.02
**SEM tooth removal**	3.8 ± 1.0 3.5 (3–6)	3.3 ± 0.9 3.0 (3–6)	0.051	0.25

† Mann–Whitney U test. Statistically significant (*p* < 0.05). ILA using CCLADS: intraligamentary anesthesia using Computer-Controlled Local Anesthesia Delivery System. IANB: inferior alveolar nerve block. SEM: Sound-Eye-Motor scale. Effect size is reported as rank-biserial correlation; positive values indicate higher scores in the IANB group compared with the ILA using CCLADS group, whereas negative values indicate higher scores in the ILA using CCLADS group.

## Data Availability

The datasets used and/or analyzed during the current study are available from the corresponding author upon reasonable request, as the dataset contains participant-level research data collected from human subjects. Although all data have been anonymized, unrestricted public sharing was not included in the participant consent and is subject to institutional ethical considerations.

## Abbreviations

The following abbreviations are used in this manuscript:

ILA	Intraligamentary anesthesia
CCLLADs	Computer-Controlled Local Anesthetic Delivery System
WBFPRS	Wong–Baker Faces Pain Rating Scale
LA	Local Anesthesia
STA	Single Tooth Anesthesia
SEM	Sound-Eye-Motor
